# Kidney Transplantation Improves Survival in Antineutrophil Cytoplasmic Antibody–Associated Vasculitides With End-Stage Kidney Disease

**DOI:** 10.1016/j.ekir.2025.02.001

**Published:** 2025-02-07

**Authors:** Benoît Brilland, Jean-François Augusto, Thomas Jouve, Noémie Jourde-Chiche, Cécile Couchoud, Renal Epidemiology and Information Network (REIN) registry

**Affiliations:** 1 Service de Néphrologie-Dialyse-Transplantation, CHU Angers, Angers, France; 2 CRCI2NA, SFR ICAT, Univ Angers, Nantes Université, Inserm, CNRS, Angers, France; 3 Nephrology, Hemodialysis, Apheresis and Kidney Transplantation Department, University Hospital Grenoble, Grenoble, France; 4 Centre de recherche en CardioVasculaire et Nutrition (C2VN), Aix-Marseille Université, INSERM, INRAE, AP-HM Centre de Néphrologie et Transplantation Rénale, Marseille, France; 5 Coordination nationale REIN, Agence de la biomédecine, Saint-Denis-La Plaine, France

**Keywords:** ANCA, death, glomerulonephritis, kidney transplantation, survival, vasculitis

## Abstract

**Introduction:**

Antineutrophil cytoplasmic antibody (ANCA)-associated vasculitis (AAV) frequently leads to end-stage kidney disease (ESKD). Although kidney transplantation (KT) is considered the optimal treatment for ESKD, its survival benefit in patients with AAV remains understudied. This study aimed to determine the impact of KT on survival in waitlisted patients with AAV-induced ESKD (AAV-ESKD).

**Methods:**

We conducted a retrospective analysis of patients with AAV-ESKD registered in the French Renal Epidemiology and Information Network (REIN) registry and waitlisted for KT between 2002 and 2022. KT was treated as a time-dependent variable to avoid immortal time bias. Survival was assessed using Kaplan-Meier analysis and Cox proportional hazards models, adjusting for key demographic and clinical factors. Subgroup analyses were conducted based on vasculitis type, age, sex, and year of ESKD onset.

**Results:**

Of 1165 patients with AAV-ESKD, 468 (40%) were waitlisted, and 318 of these (68%) received a transplant. After a median follow-up of 61 months after waitlisting, KT was associated with a 53% reduction in mortality risk (adjusted hazard ratio [HR] = 0.47 [0.31–0.73], *P* < 0.001). This benefit was consistent across subgroups. Patient survival at 10 years was 72% for transplant recipients versus 28% for nontransplanted patients (*P* < 0.001). Sensitivity analyses, after excluding recipients of living donors and patients removed from the waitlist supported the robustness of these findings. Within 2 years from ESKD onset, 24% of waitlisted patients were transplanted. Graft failure probability was 22% at 10 years posttransplant.

**Conclusion:**

KT is associated with a significant survival benefit in waitlisted patients with AAV-ESKD compared with waiting on dialysis. These findings emphasize the importance of timely transplant evaluation and improved access to KT for this population.

## Graphical abstract

**Figure:**
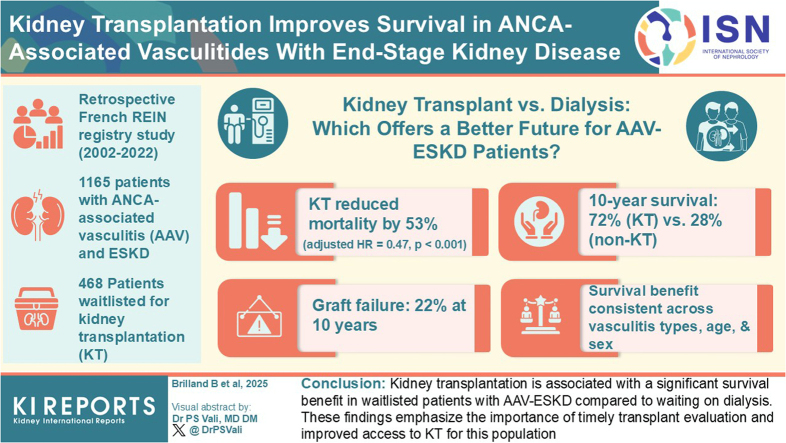

Autoimmune diseases affecting the kidneys are typically characterized by glomerulonephritis (GN), which can lead to a rapid decline in renal function and result in ESKD. Considering all causes combined, GN accounts for up to 20% of chronic kidney diseases in industrialized countries, representing a major burden on healthcare systems.^1^

AAV are among the most aggressive forms of GN.^2^ Kidney involvement, characterized by necrotizing crescentic GN, is the most frequent organ involvement in granulomatosis with polyangiitis (GPA) and microscopic polyangiitis (MPA)—the 2 main AAV phenotypes— affecting up to 80% to 95% of patients.^3^, ^4^, ^5^ AAV-associated GN (AAV-GN) has a major impact on renal and global survival^6^^,^^7^; around 25% of patients will require kidney replacement therapy within 5 years from diagnosis,^6^^,^^8^ and the estimated excess mortality is 2 to 3 times higher than that of the general population, conferring a risk of death of 20–40% at 5 years.^9^

KT has been found to be safe and successful in the AAV-GN population with ESKD.^10^^,^^11^ Athough the overall patient and graft survival appear similar for these patients when compared with other transplant recipients,^12^, ^13^, ^14^ they may have a heightened risk for some posttransplant complications, such as infections, cancer, and cardiovascular events, all of which are partly attributed to extensive pretransplant immunosuppression. Moreover, though the risk of AAV relapse decreases after dialysis initiation^15^, ^16^, ^17^ and transplantation,^18^, ^19^, ^20^, ^21^ it is not fully suppressed. These factors make it difficult to extrapolate the results of transplantation seen in all nephropathies to this specific population.

Indeed, as in other nephropathies, KT is considered the best option for overall survival compared with patients with AAV maintained on dialysis. However, the survival benefit of KT among patients with AAV-GN–induced ESKD has not been well-studied.^19^^,^^22^ In line with the results of the main studies performed in the all-cause ESKD population, demonstrating the survival benefit of transplantation,^23^^,^^24^ a previous study found that KT was associated with a 70% reduction in the risk of death among patients with GPA with ESKD waitlisted for a KT.^22^ Here, we used a national registry of patients with AAV-ESKD to determine the impact of KT on survival.

## Methods

### Data Collection

REIN is a comprehensive national registry of patients who start kidney replacement therapy in France.^25^^,^^26^ Data are collected locally by each medical center and are updated prospectively annually with the help of research assistants. The collection of data was initiated in 2002, and patients were followed-up until KT, death, loss of follow-up, recovery of kidney function, or end of the study in December 2022, whichever occurred first.

### Selection of Patients

To assess the impact of transplantation on survival, this national multicenter study included all patients who fulfilled the following criteria: (i) ESKD attributed to AAV (whether GPA or MPA); (ii) initiated hemodialysis or peritoneal dialysis (or were preemptively transplanted) between January 1, 2002, and December 31, 2022; and (iii) waitlisted for a renal transplant between January 1, 2002, and December 31, 2022. Other small vessel vasculitides, such as antiglomerular basement membrane disease, eosinophilic GPA (Churg-Strauss syndrome), and IgA vasculitis (Henoch-Schoenlein purpura) were excluded. Only the first transplant event was considered.

### Covariates and Definitions

The following information was extracted from the REIN registry^27^ and used as covariates, exposures, or outcomes: type of vasculitis (GPA or MPA); demographics (age, sex); body mass index; calculated panel reactive antibody; cardiovascular comorbidities (i.e., heart failure, heart rhythm disorders, peripheral arterial disease, abdominal aorta aneurysm, coronary artery disease, and stroke); other relevant comorbidities (i.e., diabetes, respiratory insufficiency, or cancer); initial kidney replacement therapy modality (hemodialysis or peritoneal dialysis); waitlisting date (as well as removal from the waitlist and its underlying cause when applicable); transplant status; date and type (live or deceased donor) of kidney transplant; status at the end of follow-up, and when applicable, the date and cause of death. Kidney allograft survival was also assessed, and causes of allograft loss were retrieved. Given the various numbers of cardiovascular comorbidities, they were grouped together, and the patients were stratified into 3 subgroups (no cardiovascular comorbidity, only 1, and at least 2).

### Statistical Analysis

Continuous variables were described with mean ± SD, except for delays between events, presented as median (first–third quartiles). Categorical variables were described with counts and percentages. Data were compared using *t* test for continuous variables and χ^2^ test (or Fisher exact test if necessary) for categorical variables.

Our primary outcome was all-cause mortality. The exposure of interest was the first kidney transplant. The date on which the patient was first waitlisted for a kidney transplant was used as the start of follow-up. If waitlisting occurred before dialysis started, this latter timepoint was considered as the start of follow-up.

We assessed transplantation as a time-dependent exposure, allocating time spent before a renal transplant to the group that did not have transplants and time spent after to the group that did.^22^^,^^28^ This time-dependent approach avoids immortal time bias.^29^ Transplanted patients who experienced graft failure were censored at the time of return to dialysis. Kaplan-Meier analysis was performed to estimate patient survival, and survival curves were compared using the log-rank test. Mortality rates (per 1000 patient-years) were determined by dividing the number of events during the total follow-up by the total follow-up (in years) × 1000. Mortality rate comparisons and 95% confidence intervals were estimated using the Poisson method.

Cox proportional hazards regression analysis was performed to examine factors associated with the occurrence of death. Multivariate Cox regression analysis included all parameters with *P* < 0.1 in the univariate analysis. To simplify the multivariable models, the number of variables in the multivariate Cox analysis was limited using manual step-by-step backward selection with a removal criterion of *P* > 0.1. HR with 95% confidence intervals were reported. In addition, to assess the evolution of relative risk over time, we employed a cumulative time-window analysis approach. Separate Cox models were fit for cumulative 3-month increases in follow-up (e.g., baseline to month 3, baseline to month 6, etc.) over a total period of 15 years. Follow-up time beyond each time point was censored. This method provides a dynamic estimation of the HR, thus reflecting potential variations in the association of transplantation with the risk of death over time. Again, 95% confidence intervals were calculated for each estimate.

Lastly, to assess differential results across subgroups, we calculated *P*-values for interaction. Subgroup analyses were performed to evaluate differences in all-cause mortality with and without transplantation according to sex, type of vasculitis (GPA or MPA), age group at ESKD onset (below or above 60 years) and year of ESKD onset (before or after 2012, year corresponding to the median of total follow-up in waitlisted cohort).

The probabilities of access to the waitlist, access to transplantation, and graft failure were evaluated with death as a competing event, using the cumulative incidence competing risk method.^30^ Cumulative incidence curves were compared using Gray’s test.

No imputation of missing data was performed. Statistical analyses were performed using R v4.3 with the survival, survminer and tidycmprsk packages. All tests were 2-sided, and a *P*-value < 0.05 was considered statistically significant.

### Ethical Issues

The national ethics committee (Commission National Informatique et Liberté) approved the data collection conducted by REIN, and this study was approved by the scientific committee of REIN. No informed consent was required to participate in the REIN registry. Data collection is ruled with an implicit consent with a drop-out option. It was conducted according to the principles of the Declaration of Helsinki and its amendments. The clinical and research activities reported are consistent with the Principles of the Declaration of Istanbul as outlined in the 'Declaration of Istanbul on Organ Trafficking and Transplant Tourism.

## Results

### Baseline Characteristics of all Patients With AAV-ESKD

**Figure 1:**
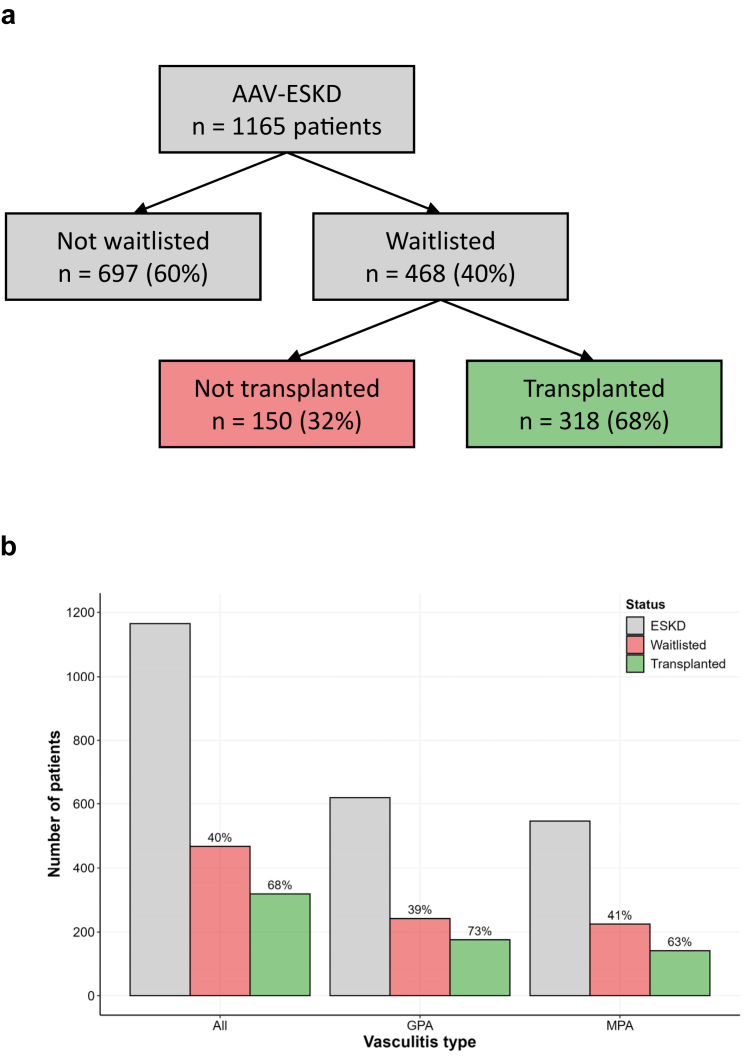
Study participants. (a) Flowchart of the study. (b) Distribution of the number of ESKD, waitlisted, and transplanted patients considering all vasculitis subtypes or each one separately. The percentages above the bars for the "waitlisted" and "transplanted" categories indicate the proportion of patients relative to the previous category (waitlisted among ESKD; transplanted among waitlisted). AAV, ANCA-associated vasculitides; ANCA, antineutrophil cytoplasmic antibody; ESKD, end-stage kidney disease; GPA, granulomatosis with polyangiitis; MPA, microscopic polyangiitis.

Between January 1, 2002 and December 31, 2022, 1165 patients were diagnosed with ESKD attributed to AAV and registered in the REIN Registry as follows: 619 (53%) GPA and 546 (47%) MPA. Overall, 468 of 1165 (40%) were waitlisted for a kidney transplant during the study period, among whom 318 of 468 (68%) subsequently underwent KT during follow-up (Figure 1). Patients with AAV-ESKD were mainly male (60%) with a mean age of 67 ± 14 years at dialysis initiation, most of them being enrolled in hemodialysis (91%) rather than peritoneal dialysis (7.5%) or preemptive transplantation (1.4%).

Compared with patients with AAV-ESKD who were not waitlisted, waitlisted patients were younger (aged 57 ± 13 vs. 74 ± 11 years at dialysis initiation) and had fewer comorbidities (Table 1). Specific data regarding each vasculitis subtype is shown in Supplementary Table S1.

**Table 1:** Baseline characteristics and outcomes of patients with AAV-ESKD

Characteristics	ESKD *n* = 1165	ESKD not waitlisted *n* = 697	ESKD waitlisted *n* = 468	*p*-value^a^	Waitlisted not transplanted *N* = 150	Waitlisted transplanted *N* = 318	Missing values	*P*-value^b^
Baseline characteristics, *n* (%)								
Vasculitis subtype				0.4			0 (0%)	0.022
GPA	619 (53%)	377 (54%)	242 (52%)		66 (44%)	176 (55%)		
MPA	546 (47%)	320 (46%)	226 (48%)		84 (56%)	142 (45%)		
Male	703 (60%)	397 (57%)	306 (65%)	0.004	103 (69%)	203 (64%)	0 (0%)	0.3
Age								
at ESKD (yrs)	67 (14)	74 (11)	57 (13)	<0.001	60 (10)	56 (14)	0 (0%)	<0.001
at waitlisting (yrs)	58 (13)	-	58 (13)		61 (10)	57 (14)	0 (0%)	<0.001
at transplantation (yrs)	59 (14)	-	59 (14)		-	59 (14)	0 (0%)	
First modality				<0.001			0 (0%)	0.014
Hemodialysis	1,062 (91%)	657 (94%)	405 (87%)		137 (91%)	268 (84%)		
Peritoneal dialysis	87 (7.5%)	40 (5.7%)	47 (10%)		13 (8.7%)	34 (11%)		
Preemptive transplantation	16 (1.4%)	0 (0%)	16 (3.4%)		0 (0%)	16 (5.0%)		
Calculated Panel Reactive Antibody (%)	-	-	14 (28)		18 (32)	12 (25)	65 (14%)	0.086
Comorbidities								
Not able to walk alone	122 (12%)	114 (18%)	8 (1.9%)	<0.001	3 (2.2%)	5 (1.8%)	48 (10%)	0.7
Albumin level < 30 g/l	417 (42%)	270 (45%)	147 (37%)	0.011	53 (40%)	94 (35%)	68 (15%)	0.4
Albumin level < 36 g/l	766 (76%)	497 (83%)	269 (67%)	<0.001	99 (74%)	170 (64%)	68 (15%)	0.045
BMI				0.013			48 (10%)	0.081
< 18.5 kg/m^2^	84 (8.4%)	53 (9.1%)	31 (7.4%)		11 (8.2%)	20 (7.0%)		
18.5–25 kg/m^2^	542 (54%)	333 (57%)	209 (50%)		56 (42%)	153 (53%)		
> 25 kg/m^2^	377 (38%)	197 (34%)	180 (43%)		67 (50%)	113 (40%)		
Diabetes mellitus	227 (20%)	157 (23%)	70 (15%)	0.002	34 (23%)	36 (12%)	8 (1.7%)	0.001
Cardiovascular comorbidities				<0.001			10 (2.1%)	0.029
None	696 (61%)	343 (50%)	353 (77%)		103 (70%)	250 (80%)		
Only 1	287 (25%)	208 (30%)	79 (17%)		31 (21%)	48 (15%)		
At least 2	163 (14%)	137 (20%)	26 (5.7%)		13 (8.8%)	13 (4.2%)		
Respiratory insufficiency	195 (17%)	143 (21%)	52 (11%)	<0.001	28 (19%)	24 (7.7%)	11 (2.4%)	<0.001
Cancer	66 (5.9%)	50 (7.4%)	16 (3.6%)	0.01	5 (3.4%)	11 (3.7%)	28 (6.0%)	0.9
Timings and delays^c^								
Time from dialysis to waitlisting (mo)	-	-	13 (5, 23)	-	15 (7, 26)	13 (4, 21)	0 (0%)	0.047
Time from dialysis to KT (mo)	-	-	32 (19, 47)	-	-	32 (19, 47)	0 (0%)	-
Time from waitlisting to KT (mo)	-	-	15 (6, 27)	-	-	15 (6, 27)	0 (0%)	-
Follow-up since dialysis initiation (mo)	51 (20, 95)	35 (11, 71)	80 (43, 123)	<0.001	42 (21, 68)	102 (62, 143)	0 (0%)	<0.001
Follow-up since waitlisting (mo)	-	-	61 (26, 107)	-	20 (4, 46)	83 (49, 126)	0 (0%)	<0.001
Follow-up since transplantation (mo)	-	-	63 (20, 108)	-	-	63 (20, 108)	0 (0%)	-
Transplantation								
Donor type				-			0 (0%)	-
None	-	697 (100%)	150 (32%)		150 (100%)	-		
Deceased donor	-	-	282 (60%)		-	282 (89%)		
Living donor^d^	-	-	36 (7.7%)		-	36 (11%)		
Waitlist removal^d^	-	-	34 (7.3%)	-	34 (23%)	-	0 (0%)	-
Outcomes								
Graft failure	50 (16%)	-	50 (16%)	-	-	50 (16%)	0 (0%)	-
Death	656 (56%)	508 (73%)	148 (32%)	<0.001	62 (41%)	86 (27%)	0 (0%)	0.002
During waitlisted period	-	-	-	-	44 (71%)	-	0 (0%)	-
After waitlist removal^d^	-	-	-	-	18 (29%)	-	0 (0%)	-
During transplant period	-	-	-	-	-	63 (73%)	0 (0%)	-
After return to dialysis^e^	-	-	-	-	-	23 (27%)	0 (0%)	-

AAV-ESKD, ANCA-associated vasculitis with end-stage kidney disease; ANCA, antineutrophil cytoplasmic antibody; BMI, body mass index; ESKD, end-stage kidney disease; GPA, granulomatosis with polyangiitis; KT, kidney transplantation; MPA, microscopic polyangiitis.

“-” indicates not applicable.

a Comparison between patients with ESKD on the waitlist and those not on the waitlist.

b Comparison between waitlisted not transplanted and waitlisted transplanted patients.

c Timing between events and delays are shown as median [first to third quartile], in opposition to other continuous data, shown as mean (SD).

d Patients excluded from the sensitivity analyses.

e Patients were censored from subsequent survival analyses.

### Baseline Characteristics of Waitlisted Patients With AAV

Most waitlisted patients were male (65%) with a mean age of 58 ± 13 years at the time of waitlisting for KT. The median duration of dialysis before waitlisting was 13 (5–23) months. Regarding the comorbidities, 23% of patients had at least 1 cardiovascular comorbidity, 15% were diabetic, and 11% had respiratory insufficiency. Most patients could walk alone (98%), and many had albumin levels below 30 g/l (37%). Lastly, 43% had a body mass index > 25 kg/m^2^ and 7% below 18.5 kg/m^2^.

Compared with waitlisted patients who were not transplanted during the study period, patients who received a kidney transplant were younger (57 ± 14 vs. 61 ± 10 years old at the time of transplantation), with fewer comorbidities. Most transplanted patients were male (64%) and GPA (55%) with a mean age at the time of KT of 59 ± 14 years. The median waitlist duration was 15 (6-27) months. Transplanted patients tended to have lower calculated panel reactive antibody(12 ± 25 vs. 18 ± 32). Deceased donor transplantation was performed in most cases (89%). During follow-up, 34 patients (23%) were removed from the waitlist before being transplanted (Table 1). Their characteristics are described in Supplementary Table S2; they were older and more likely to be diabetic. Reasons for waitlist removal are detailed in Supplementary Table S3). Specific data regarding each vasculitis subtype is shown in Supplementary Table S1.

### Causes of Death in AAV Waitlisted Patients

After a median follow-up of 61 (26–107) months since waitlisting, 148 patients (32%) died, the details are as follows: 86 (27%) who received a kidney transplant (including 63 [76%] during the transplant period and 23 [27%] after return to dialysis) and 62 (41%) who did not (including 44 [71%] during the waitlisted period and 18 [29%] after being removed from the waitlist) (Table 1). The cause of death was not known in 26 patients (18%) and labelled as “others causes” in 26 patients (18%). The proportion of death from infectious causes was similar in both groups (26% vs. 27% in the nontransplanted vs. transplanted group), the proportion of death from cancer causes was not significantly higher in the transplanted group (19% vs. 15%), and the proportion of death from cardiovascular causes was not significantly lower in the transplanted group (12 vs. 15%) (Supplementary Table S4). Causes of death for nonwaitlisted patients and according to the moment of death in waitlisted patients are also available in Supplementary Table S4).

### All-Cause Mortality in Waitlisted Patients With AAV

The mortality rate among those who received a kidney transplant was 38.6 (29.1–48.1)per 1000 patient-years, in contrast to 67.2 (50.5–84.0] per 1000 patient-years among those who did not receive kidney transplant. Such a reduction in mortality was consistent across all subgroups (*P* = 0.002, Table 2).

**Table 2:** All-cause mortality according to transplant status among waitlisted patients with AAV-ESKD

Characteristics	*n*	Total follow-up (yrs)	Deaths	Mortality rate (/1000 person-yrs)	Mortality rate 95% CI	Unadjusted			Adjusted on age/sex			Fully adjusted^a^		
						HR	HR 95% CI	*P*-value for interaction	HR	HR 95% CI	*P*-value for interaction	HR	HR 95% CI	*P*-value for interaction
Overall	786	2.554	125	48.94	40.36–57.51									
Dialysis, waitlisted	468	922	62	67.24	50.50–83.98	—	—		—	—		—	—	
Transplanted	318	1.632	63	38.59	29.06–48.13	0.38	0.25–0.56		0.39	0.26–0.58		0.47	0.31–0.73	
Age (waitlisting)								0.50			0.60			0.49
> 60 yrs	405	1.153	82	71.11	55.72–86.50									
Dialysis, waitlisted	249	483	42	86.88	60.61–113.16	—	—		—	—		—	—	
Transplanted	156	670	40	59.72	41.21–78.23	0.41	0.24–0.70		0.41	0.24–0.70		0.51	0.30–0.89	
≤ 60 yrs	381	1.401	43	30.69	21.52–39.86									
Dialysis, waitlisted	219	439	20	45.60	25.61–65.58	—	—		—	—		—	—	
Transplanted	162	963	23	23.89	14.13–33.66	0.31	0.16–0.60		0.29	0.14–0.59		0.36	0.17–0.81	
Sex								0.59			0.62			0.56
Female	277	961	39	40.58	27.85–53.32									
Dialysis, waitlisted	162	335	21	62.69	35.88–89.50	—	—		—	—		—	—	
Transplanted	115	626	18	28.75	15.47–42.04	0.39	0.20–0.76		0.38	0.19–0.77		0.53	0.25–1.14	
Male	509	1.593	86	53.97	42.57–65.38									
Dialysis, waitlisted	306	587	41	69.84	48.46–91.22	—	—		—	—		—	—	
Transplanted	203	1.006	45	44.72	31.65–57.78	0.33	0.19–0.55		0.36	0.21–0.61		0.47	0.28–0.81	
Vasculitis subtype								0.99			0.56			0.56
GPA	418	1.507	66	43.81	33.24–54.38									
Dialysis, waitlisted	242	470	28	59.55	37.49–81.61	—	—		—	—		—	—	
Transplanted	176	1.036	38	36.67	25.01–48.33	0.37	0.21–0.64		0.36	0.21–0.65		0.42	0.23–0.77	
MPA	368	1.048	59	56.30	41.94–70.67									
Dialysis, waitlisted	226	452	34	75.24	49.95–100.53	—	—		—	—		—	—	
Transplanted	142	596	25	41.95	25.50–58.39	0.41	0.23–0.72		0.47	0.26–0.83		0.53	0.29–0.97	
Date of KRT start								0.19			0.10			0.19
Before 2012	262	1.309	63	48.14	36.25–60.03									
Dialysis, waitlisted	142	274	22	80.24	46.71–113.78	—	—		—	—		—	—	
Transplanted	120	1.034	41	39.63	27.50–51.77	0.34	0.19–0.60		0.31	0.17–0.57		0.41	0.21–0.82	
After 2012	524	1.246	62	49.77	37.38–62.16									
Dialysis, waitlisted	326	648	40	61.74	42.61–80.87	—	—		—	—		—	—	
Transplanted	198	598	22	36.80	21.42–52.17	0.46	0.27–0.79		0.48	0.28–0.84		0.60	0.34–1.05	

AAV-ESKD, ANCA-associated vasculitis with end-stage kidney disease; ANCA, antineutrophil cytoplasmic antibody; CI, confidence interval; ESKD, end-stage kidney disease; GPA, granulomatosis with polyangiitis; HR, hazard ratio; KRT, kidney replacement therapy; MPA, microscopic polyangiitis.

Total follow-up was computed from time zero (time which patients entered their respective risk groups [not the cohort entry or transplant date]) to end of follow-up. Time spent before kidney transplantation was allocated to the “dialysis, waitlisted” group and time spent after was allocated to the “transplanted” group. This implies that the initial total “dialysis, waitlisted” group size will be equal to the “dialysis, waitlisted” group size + “transplanted” group size.

a Fully adjusted models were adjusted for age at waitlisting, sex (forced), vasculitis type, cardiovascular comorbidity, and respiratory insufficiency (Table 3).

**Figure 2:**
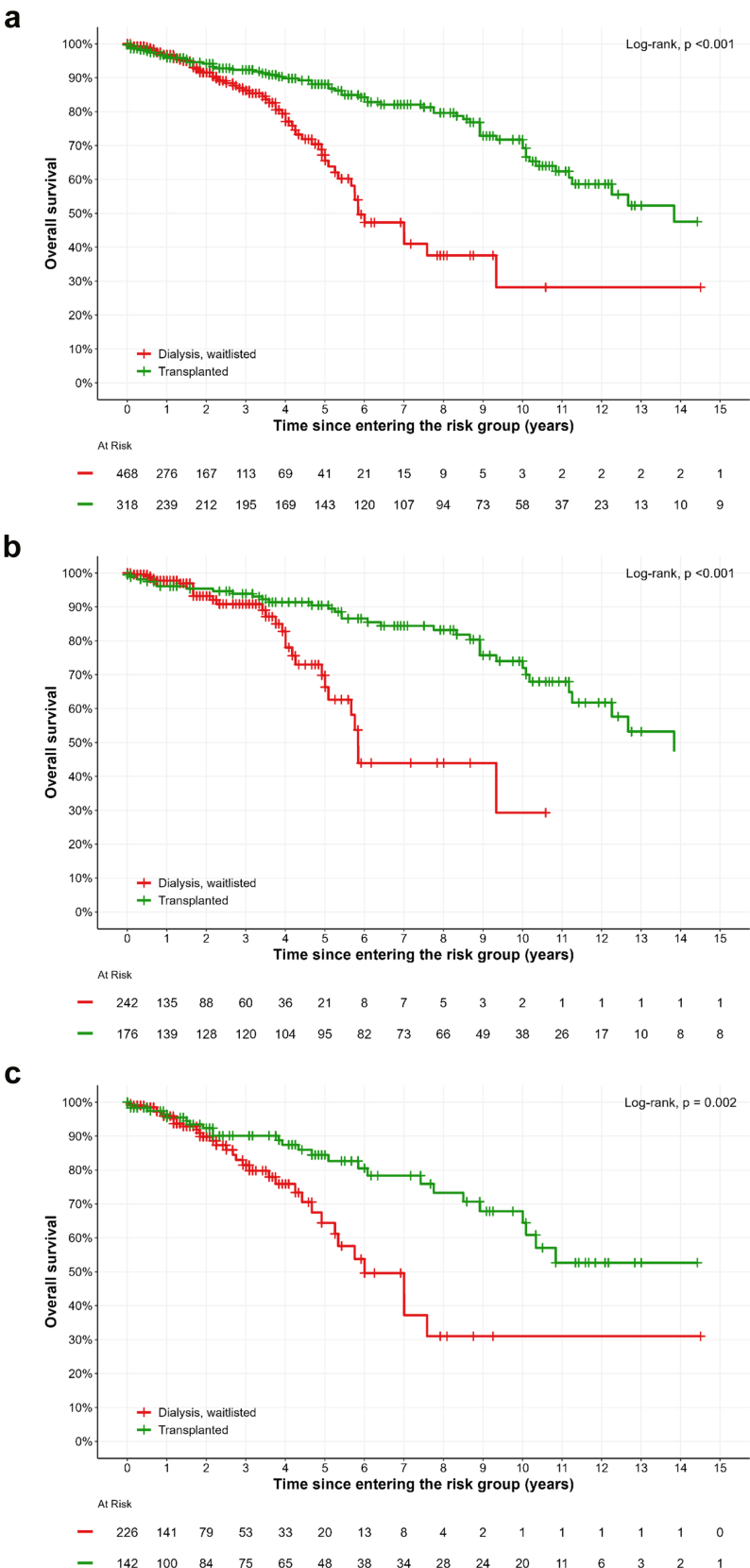
Patient survival according to transplant status among waitlisted patients with AAV-ESKD. (a) All vasculitis subtypes. (b) GPA. (c) MPA. Time zero represents the point at which patients entered their respective risk groups (not the cohort entry or transplant date). Time spent before kidney transplantation was allocated to the “dialysis, waitlisted” group and time spent after was allocated to the “transplanted” group. This implies that the initial total “dialysis, waitlisted” group size will be equal to the “dialysis, waitlisted” group size + “transplanted” group size. GPA, granulomatosis with polyangiitis; MPA, microscopic polyangiitis.

In the cohort of waitlisted patients with AAV, patient survival was 88% (84%–92%) and 72% (65%–80%) at 5 and 10 years, respectively, in transplant recipients, in contrast to 67% (59%–77%) and 28% (14%–55%) at 5 and 10 years, respectively, in nontransplanted patients. The median patient survival was 13.8 years for transplant recipients versus 5.8 years for nontransplanted patients (*P* < 0.001, Figure 2a). This improved survival was found consistently in GPA (*P* < 0.001, Figure 2b) and MPA (*P* = 0.002, Figure 2c) subgroups. Survival from ESKD onset (start of dialysis) for nonwaitlisted or nontransplanted patients is shown in Supplementary Figure S1).

### Risk of Death in Waitlisted Patients With AAV

Factors associated with death in univariable analyses are presented in Table 3. Age at waitlisting (HR = 1.06 [1.04–1.08] per 1 year increment, *P* < 0.001), MPA vasculitis subtype (vs. GPA, HR = 1.44 [1.01–2.06], *P* = 0.046), inability to walk alone (HR = 3.46 [1.27–9.44], *P* = 0.015), cardiovascular comorbidities (1 vs. none, HR = 1.46 [0.92–2.29, *P* = 0.11; ≥ 2 vs. none, HR = 2.51 [1.33–4.72], *P* = 0.004), and respiratory insufficiency (HR = 3.40 [2.14–5.40], *P* < 0.001) were associated with death. KT (HR = 0.38 [0.25–0.56], *P* < 0.001) was associated with survival.

**Table 3:** Factors associated with death among waitlisted patients with AAV-ESKD

Characteristics	Univariable analysis					Multivariable analysis				
	*n*	Deaths	HR	95% CI	*P*-value	*n*	Deaths	HR	95% CI	*P*-value
Baseline characteristics										
Vasculitis subtype										
GPA	418	66	—	—		404	62	—	—	
MPA	368	59	1.44	1.01–2.06	0.046	363	56	1.32	0.91–1.93	0.15
Males	509	86	1.41	0.96–2.07	0.077	494	81	1.30	0.87–1.92	0.2
Age (per 1 year increment)										
at ESKD (yrs)	786	125	1.06	1.04–1.08	<0.001					
at waitlisting (yrs)	786	125	1.06	1.04–1.08	<0.001	767	118	1.06	1.03–1.08	<0.001
at transplantation (yrs)	318	63	1.07	1.04–1.10	<0.001					
First modality										
Hemodialysis	673	115	—	—						
Peritoneal dialysis	81	7	0.46	0.21–0.99	0.046					
Preemptive transplantation	32	3	0.53	0.17–1.67	0.3					
Year of ESKD: before 2012	262	63	0.68	0.45–1.02	0.061					
cPRA (per 1% increment)	662	97	1	0.99–1.01	0.9					
Comorbidities										
Not able to walk alone	13	4	3.46	1.27–9.44	0.015					
Albumin level > 30 g/l	425	58	0.69	0.46–1.03	0.071					
Albumin level > 36 g/l	227	29	0.70	0.46–1.09	0.11					
BMI										
18.5–25 kg/m^2^	362	48	—	—						
< 18.5 kg/m^2^	51	8	0.84	0.39–1.79	0.6					
> 25 kg/m^2^	293	50	1.23	0.82–1.83	0.3					
Diabetes mellitus	772	121	1.42	0.84–2.40	0.2					
Cardiovascular comorbidities										
None	603	85	—	—		601	83	—	—	
Only 1	127	24	1.46	0.92–2.29	0.11	127	24	0.96	0.60–1.53	0.9
At least 2	39	11	2.51	1.33–4.72	0.004	39	11	1.55	0.80–3.00	0.2
Respiratory insufficiency	769	118	3.40	2.14–5.40	<0.001	767	118	2.34	1.45–3.78	<0.001
Cancer	734	116	1.36	0.50–3.71	0.5					
Outcomes										
Waitlist removal^a^	786	125	2.77	1.67–4.61	<0.001					
Transplantation (vs. none)	318	63	0.38	0.25–0.56	<0.001	311	61	0.47	0.31–0.73	<0.001
Donor type^a^										
None	468	62	—	—						
Deceased donor	282	59	0.39	0.26–0.59	<0.001					
Living donor	36	4	0.23	0.08–0.63	0.004					

AAV-ESKD, ANCA-associated vasculitis with end-stage kidney disease; BMI, body mass index; CI, confidence interval; cPRA, calculated panel reactive antibody; ESKD, end-stage kidney disease; GPA, granulomatosis with polyangiitis; HR, hazard ratio; KT, kidney transplantation; MPA, microscopic polyangiitis.

a Not included in the multivariate analysis. See sensitivity analysis.

After adjustment on age at waitlisting, sex (forced in the model), vasculitis subtype (forced in the model), and respiratory and cardiovascular comorbidities, KT was associated with a 53% reduction in the risk of death (HR = 0.47 [0.31–0.73], *P* < 0.001) (Table 2, Table 3). Of note, patients waitlisted before 2012 (vs. after 2012) were significantly younger, more frequently of GPA subtype, and less frequently diabetic (Supplementary Table S4). No data regarding treatment of vasculitis before waitlisting was available.

### Risk of Death in Subgroups of Waitlisted Patients With AAV

Our results were similar across all the subgroups analyzed, including sex, vasculitis type (GPA, or MPA), age at waitlisting (below or above 60 years), and year of ESKD onset (before or after 2012); whether adjusted on age and sex alone, or fully adjusted (age at waitlisting, sex, vasculitis subtype, respiratory and cardiovascular comorbidities), KT was associated with a reduction in the risk of death, without significant interaction. The strongest association was found for younger patients (HR = 0.36) (Table 2 and Supplementary Figure S1).

### Instantaneous Hazard of Death

When analyzing the time-dependent instantaneous hazard, after full adjustment (as described above), KT was associated with a higher risk of death during the first months after transplantation, followed by a lower risk over the remaining study period (Supplementary Figure S2).

### Sensitivity Analyses

We conducted 2 sensitivity analyses to confirm the findings of our primary analysis. First, to better address potential confounding because of the indication (or contraindication) that may arise when patients become too ill (the main cause of waitlist removal, 73.5%, Supplementary Table S2), or otherwise unsuitable for renal transplantation, we excluded patients who were removed from the waitlist during the study period (*n* = 34). In this analysis, the survival benefit associated with transplantation was diminished (adjusted HR = 0.57 [0.35–0.92], *P* = 0.023), which was anticipated because of the introduction of informative censoring (i.e., censoring related to a factor in the causal pathway between being waitlisted and mortality: those with a higher likelihood of death were selectively removed from the nontransplanted group). Second, because these patients may differ from others on the waitlist with respect to unmeasured confounding factors, we censored living donor transplant recipients (*n* = 36). This analysis revealed a similar survival benefit for KT as observed in our primary analysis (adjusted HR = 0.49 [0.32–0.76], *P* = 0.001).

### Kidney Allograft Survival

**Figure 3:**
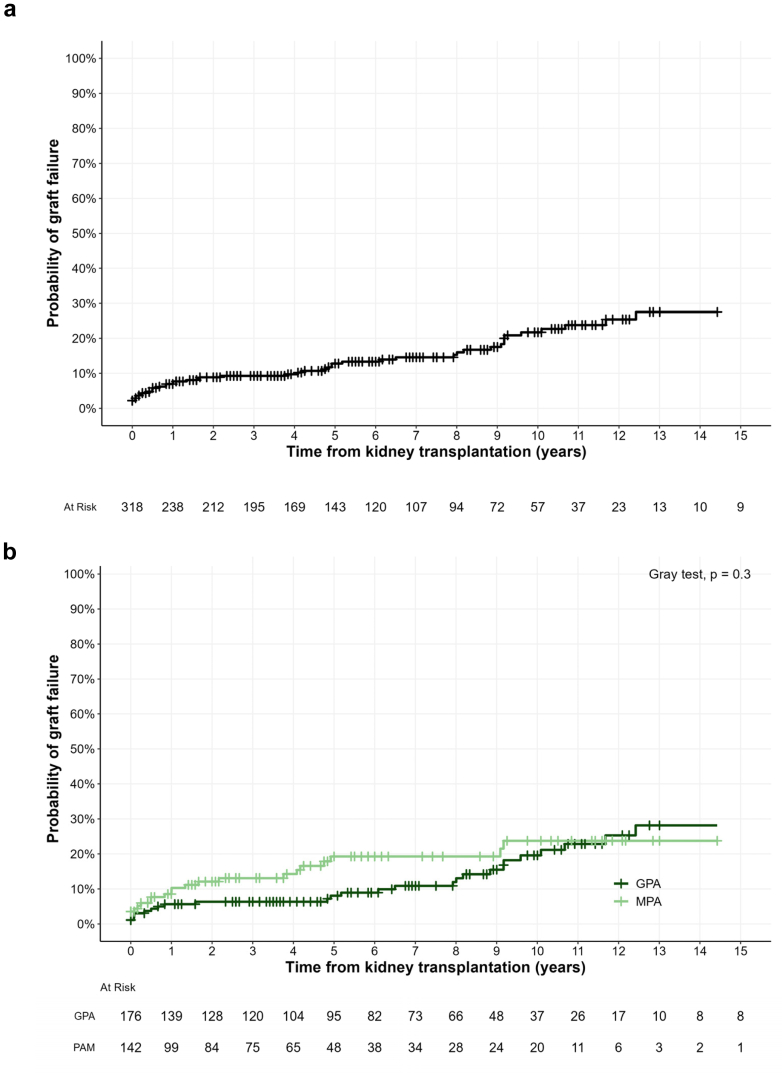
Graft survival among transplanted patients with AAV-ESKD. (a) All vasculitis subtypes. (b) According to GPA or MPA. Graft survival was computed with death as a competitive event. AAV, ANCA-associated vasculitides; ANCA, antineutrophil cytoplasmic antibody; ESKD, end-stage kidney disease; GPA, granulomatosis with polyangiitis; MPA, microscopic polyangiitis.

After a median follow-up of 63 (20–108) months since transplantation, 50 patients (16%) experienced graft failure. The main causes of allograft failure were chronic rejection (*n* = 17, 34%), followed by vascular complications (*n* = 11, 22%) and vasculitis relapses (*n* = 4, 8%) (Supplementary Table S5). The cumulative incidence of graft failure, when considering death as a competing event, was 13% (9%–17%) and 22% (16%–28%) by 5 and 10 years, respectively (Figure 3a). There was no difference in kidney allograft survival regarding the vasculitis subgroup, with an incidence of graft failure reaching 20% (12%–28%) in GPA and 24% (15%–34%) in MPA by 10 years (*P* = 0.3, Figure 3b).

### Access to Waitlist and to Transplantation

Lastly, we analyzed the probability of being waitlisted or transplanted, considering death as a competing event (Supplementary Figure 3). Among all patients with ESKD, the rates of waitlisting were 18%, 31%, and 41%, and the rates of transplantation were 4%, 10%, and 27% by 1, 2, and 5 years from ESKD onset, respectively. Among waitlisted patients, the rates of waitlisting were 43%, 76%, and 97%, and the rates of transplantation were 9%, 24%, and 66% by 1, 2, and 5 years from ESKD onset, respectively. The rates of transplantation were 31%, 52%, and 74% by 1, 2, and 5 years from waitlisting, respectively.

## Discussion

This large population study, spanning 2 decades and encompassing all French patients with AAV-ESKD who were waitlisted for KT, revealed a marked improvement in survival associated with kidney transplant. This survival benefit was consistent across key demographics, including sex and age groups throughout the study period. Notably, a considerable number of waitlisted patients died before receiving a transplant, highlighting the need to enhance kidney transplant accessibility for patients with AAV-ESKD. In addition, these findings underscore the importance of routinely evaluating patients with AAV with advanced chronic kidney disease for transplant eligibility as part of their standard care.

Our findings align with and expand on existing research on patients with AAV-ESKD and KT. The survival of patients with AAV on chronic dialysis has been shown to be similar to that of other dialysis patients,^31^, ^32^, ^33^ but with a higher rate of mortality from infectious causes. In addition, though the incidence of relapse decreases in chronic dialysis, their risk of developing an infectious or cardiovascular complication increases.^15^, ^16^, ^17^ In this context, KT may appear to be a risky option because of the additional immunosuppression required, with its known risk of infection but also diabetes or hypertension, which limits the extrapolation of kidney transplant outcomes from other ESKD patient groups to those with AAV-ESKD. However, a study conducted in the United States Renal Data System registry, using a methodology similar to ours, showed that the overall survival of patients with ESKD with GPA was better after transplantation than before transplantation (mortality rate decreasing from 65.5–29.3 deaths per 1000 patient-years, 70% reduction in the risk of death),^22^ with a benefit close to that seen in the overall ESKD population (68%–82% reduction).^23^^,^^24^ Death from infectious causes was reduced; however, the beneficial effect was even greater in reducing the risk of cardiovascular mortality. There were no data on relapse risk reduction; however, we can assume that modern immunosuppressive regimen, limiting steroid exposure, and the inflammatory state associated with the vasculitis helped to reduce the mortality associated with these events. In our cohort, we did not find such a significant reduction in death from infectious or cardiovascular causes, but similar rates, suggesting—while limited by our sample size—that prolonged immunosuppression did not increase this risk in transplanted patients when compared with patients waiting for a KT on dialysis. However, it is important to note that waitlisted patients face higher infection-related deaths (26% in our AAV-ESKD waitlisted cohort, vs. 15% in all ESKD regardless of its cause [data from REIN registry]^34^), and transplanted patients face higher cancer-related deaths (19% in our AAV-ESKD waitlisted cohort, vs. 10% in all ESKD regardless of its cause [data from REIN registry]^34^).

Notably, overall survival in transplanted patients with AAV previously appeared to be similar to that of other transplant patients,^12^, ^13^, ^14^ although there are discrepancies between studies, particularly with regard to the choice of reference group.^31^^,^^33^ Similarly, graft survival in patients with AAV appears to be close to that of other transplant recipients,^12^^,^^13^ again with some discrepancies between studies.^14^^,^^31^^,^^33^

Taken together, these data suggest that all patients with AAV-GN should be evaluated for possible inclusion on the waiting list, as soon as estimated glomerular filtration rate goes below 20 ml/min per 1.73 m^2^, as for other kidney diseases. To optimize the patient's chances of receiving a transplant under good conditions, a thorough evaluation may be necessary (nutritional, muscular, and cardiovascular evaluation, etc.). Even better, referring patients to a kidney transplant center as soon as possible may improve their chance of a preemptive transplantation, ideally with a living donor. In the worst-case scenario, patients will accrue time on the transplantation waiting list to reduce their waiting time.

Interestingly, we noted that the survival benefit associated with transplantation appeared to be higher for patients with AAV-ESKD who started dialysis before 2012 than after. This is probably explained by the fact that the criteria for waitlisting or transplantation have been expanded overtime, both at the donor and recipient level.^34^ Indeed the patients transplanted more recently in our study were older and more diabetic. In addition, changes in chronic dialysis practices have improved survival,^35^ potentially leading ultimately to a decrease in survival benefit in the latest period.

### Limits and Strengths

The strengths of this study are related to our data source and study design. First, the REIN registry is a nationwide registry covering all patients with ESKD in France. The diagnosis of AAV was made and reported by nephrologists, limiting the risk of false-positive diagnoses. We restricted our analysis to patients on the waiting list to reduce bias associated with confounding by contraindication (people on the waiting list for KT are generally younger, healthier, and with higher socioeconomic status^36^ than those who are not), and we also performed a sensitivity analysis excluding patients who were removed from the waiting list or those receiving from a living donor, and we treated KT as a time-varying exposure to limit immortal time bias. Although residual confounding can still be present, we adjusted our analysis for important factors, including comorbidities and vasculitis subgroup, to account for variables known to have a significant impact on mortality. The generalizability of these findings may be influenced by differences in waitlist criteria, patient demographics, and transplantation practices across countries and healthcare systems. Therefore, caution is needed when extrapolating these results to settings with distinct allocation policies or patient populations, because these factors could modify the observed survival benefit of KT in patients with AAV-ESKD.

Our retrospective study has limitations. First, the REIN registry enrolls patients when they reach ESKD but does not include details on the history of AAV. Therefore, we cannot address how certain factors, such as time between vasculitis onset, vasculitis remission and transplantation, antibody type and titer at diagnosis or transplantation, and exposure to immunosuppression, pretransplant events (e.g., infections, cardiovascular events, relapses) may affect outcomes. Second, during this study of a 2-decade period, substantial treatment advances have been made, especially regarding the use of rituximab for AAV induction and maintenance therapy.^37^, ^38^, ^39^

### Conclusion

In summary, in this national cohort study of patients with ESKD because of AAV, we found that KT was associated with a significant survival benefit. Timely consideration of kidney transplant should be a part of routine care for patients with AAV-ESKD, and improved access to KT for this population may considerably improve outcomes.

## Disclosure

All the authors declared no competing interests.

## Appendix

### List of Renal Epidemiology and Information Network (REIN)

Dialysis centers participating in the registry are listed in the REIN annual report (https://www.agence-biomedecine.fr/IMG/pdf/rapport_2022_-_20241128.pdf).

## Supplementary Material

Supplementary File (PDF) **Figure S1.** Patient survival according to waitlisted and transplanted status among all patients with AAV-ESKD. **Figure S2.** Fully adjusted subgroup analyses regarding the risk of death among waitlisted patients with AAV-ESKD. **Figure S3.** Instantaneous hazard of death over the study period. **Figure S4.** Cumulative incidence of being waitlisted or transplanted. **Table S1.** Baseline characteristics of waitlisted patients with AAV-ESKD according to vasculitis subtype. **Table S2.** Baseline characteristics of waitlisted patients with AAV-ESKD according to removal status during follow-up. **Table S3.** Causes of waitlist removal in AAV-ESKD waitlisted, not transplanted patients. **Table S4.** Causes of death in patients with AAV-ESKD. **Table S5.** Baseline characteristics of waitlisted patients with AAV-ESKD according to date of dialysis initiation (before or after 2012). **Table S6.** Causes of allograft failure in transplanted patients with AAV-ESKD.

## Footnotes

Supplementary File (PDF)
**Figure S1.** Patient survival according to waitlisted and transplanted status among all patients with AAV-ESKD. **Figure S2.** Fully adjusted subgroup analyses regarding the risk of death among waitlisted patients with AAV-ESKD. **Figure S3.** Instantaneous hazard of death over the study period. **Figure S4.** Cumulative incidence of being waitlisted or transplanted. **Table S1.** Baseline characteristics of waitlisted patients with AAV-ESKD according to vasculitis subtype. **Table S2.** Baseline characteristics of waitlisted patients with AAV-ESKD according to removal status during follow-up. **Table S3.** Causes of waitlist removal in AAV-ESKD waitlisted, not transplanted patients. **Table S4.** Causes of death in patients with AAV-ESKD. **Table S5.** Baseline characteristics of waitlisted patients with AAV-ESKD according to date of dialysis initiation (before or after 2012). **Table S6.** Causes of allograft failure in transplanted patients with AAV-ESKD.
