# Supplementary material for: Kidney Transplantation Improves Survival in Antineutrophil Cytoplasmic Antibody–Associated Vasculitides With End-Stage Kidney Disease
Source: Kidney Int Rep. 2025 Feb 7;10(5):1415–27. doi: 10.1016/j.ekir.2025.02.001 (PMC12142516; doi:10.1016/j.ekir.2025.02.001)
Supplement: Supplementary File (PDF) — Figure S1. Patient survival according to waitlisted and transplanted status among all patients with AAV-ESKD. Figure S2. Fully adjusted subgroup analyses regarding the risk of death among waitlisted patients with AAV-ESKD. Figure S3. Instantaneous hazard of death over the study period. Figure S4. Cumulative incidence of being waitlisted or transplanted. Table S1. Baseline characteristics of waitlisted patients with AAV-ESKD according to vasculitis subtype. Table S2. Baseline characteristics of waitlisted patients with AAV-ESKD according to removal status during follow-up. Table S3. Causes of waitlist removal in AAV-ESKD waitlisted, not transplanted patients. Table S4. Causes of death in patients with AAV-ESKD. Table S5. Baseline characteristics of waitlisted patients with AAV-ESKD according to date of dialysis initiation (before or after 2012). Table S6. Causes of allograft failure in transplanted patients with AAV-ESKD. [file mmc1.pdf]

Kidney transplantation improves survival in ANCA-associated vasculitides with end-stage kidney disease

Benoît Brilland[1,2], Jean-François Augusto[1,2], Thomas Jouve[3], Noémie Jourde-Chiche[4], Cécile Couchoud[5], on behalf of the REIN registry.

Supplementary Figures and Tables legends.

Supplementary Figure S1. Patient survival according to waitlisted and transplanted status among all patients with AAV-ESKD.

Time zero represents the point at which patients started dialysis (ESKD onset).

Supplementary Figure S2. Fully adjusted subgroup analyses regarding the risk of death among waitlisted patients with AAV-ESKD.

Fully adjusted subgroup analyses refers to analyses adjusted for age at waitlisting, sex, vasculitis type, cardiovascular comorbidity and respiratory insufficiency.

Abbreviations: AAV, ANCA-associated vasculitides; ESKD, end-stage kidney disease; GPA, granulomatosis with polyangiitis; MPA, microscopic polyangiitis.

Supplementary Figure S3. Instantaneous hazard of death over the study period.

A) Raw instantaneous hazard. B) Age and sex adjusted instantaneous hazard. C) Fully adjusted (with adjustment for age at waitlisting, sex, vasculitis type, cardiovascular comorbidity and respiratory insufficiency) instantaneous hazard.

Supplementary Figure S4. Cumulative incidence of being waitlisted or transplanted.

Cumulative incidence of accessing to waitlist or transplantation was computed with death as a competitive event. The curves represent the time from ESKD onset to transplantation (“ESKD > KT”, blue), from ESKD onset to waitlisting (“ESKD > WL”, green), and from waitlisting to transplantation (“WL > KT”, red).

Abbreviations: ESKD, end-stage kidney disease; KT, kidney transplantation; WL, waitlisting.

Sup. Figure S1

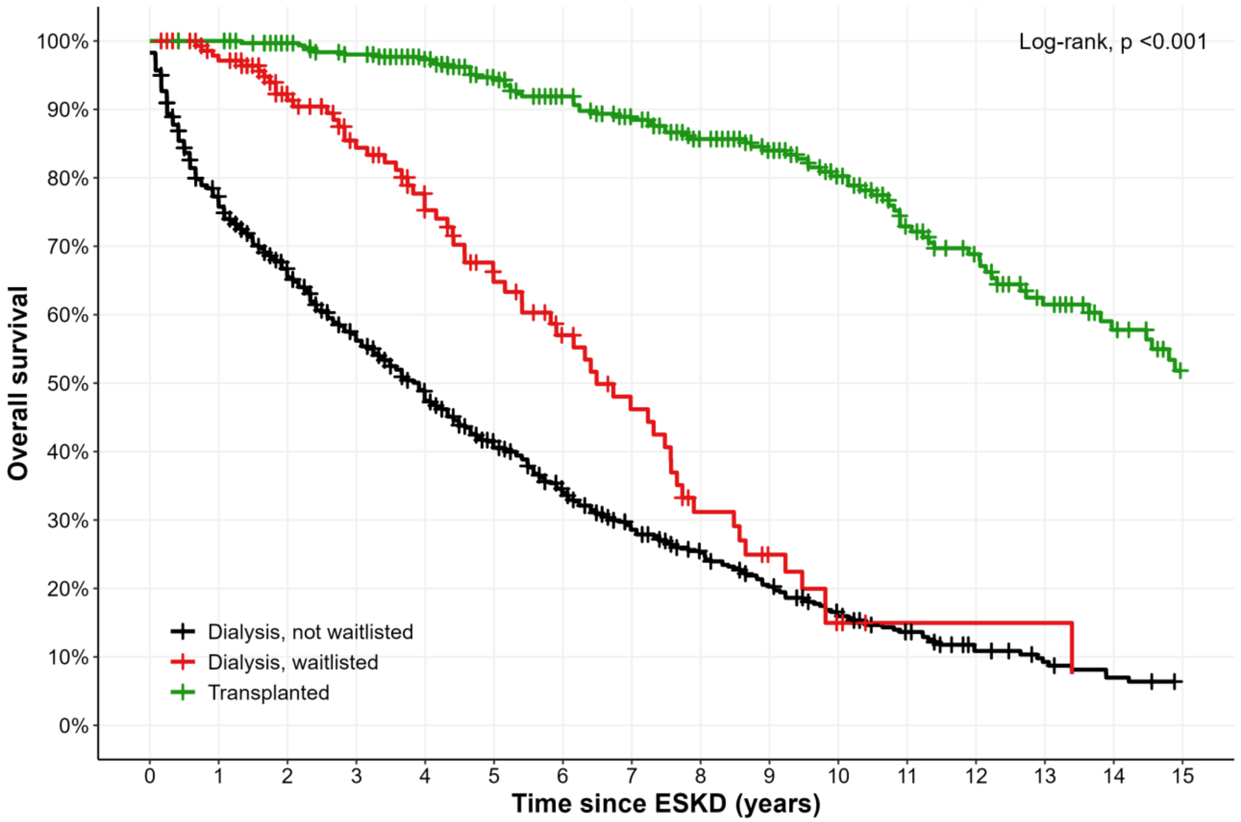

At Risk

—	697	513	414	338	270	217	166	124	100	75	52	38	24	17	12	9
—	150	134	101	80	61	44	33	25	15	10	5	2	2	2	1	1
—	318	317	303	291	267	241	218	199	168	146	120	94	79	61	46	32

Sup. Figure S2

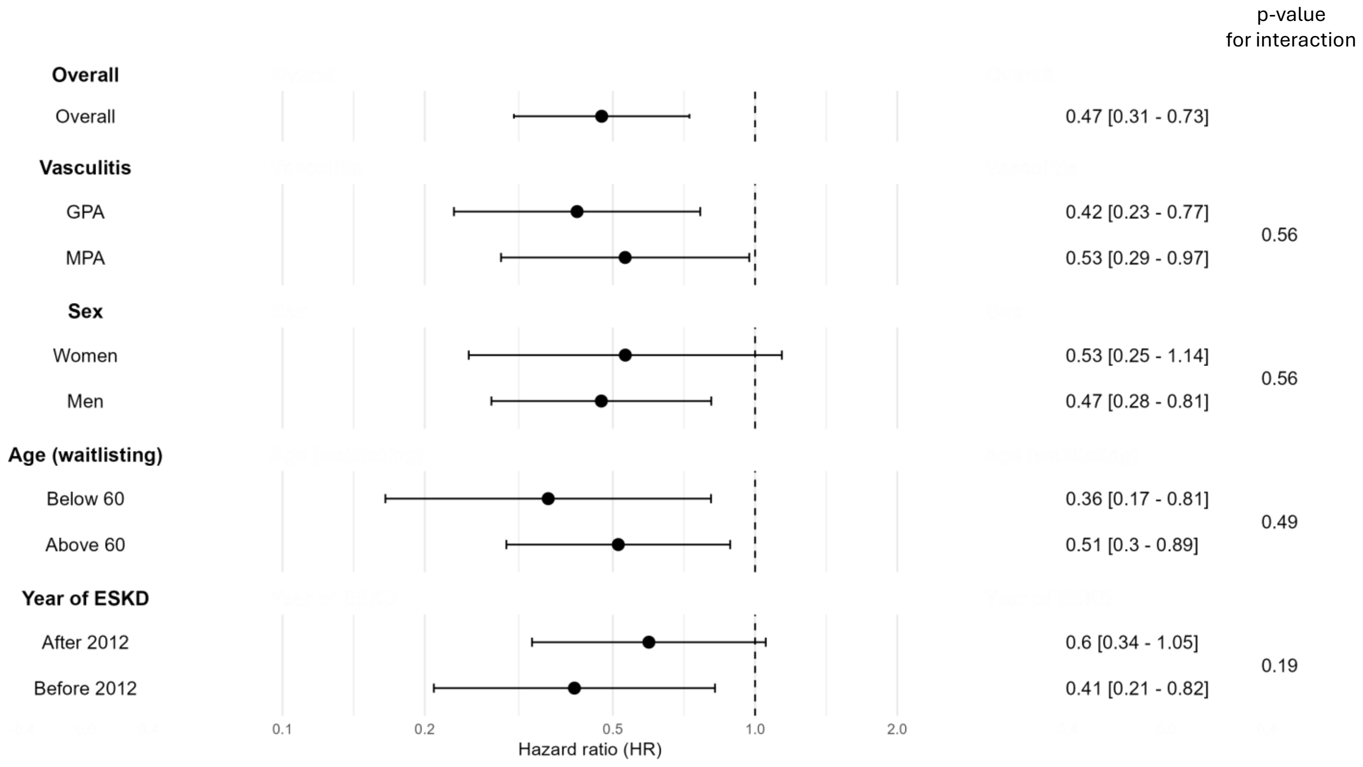

Sup. Figure 3

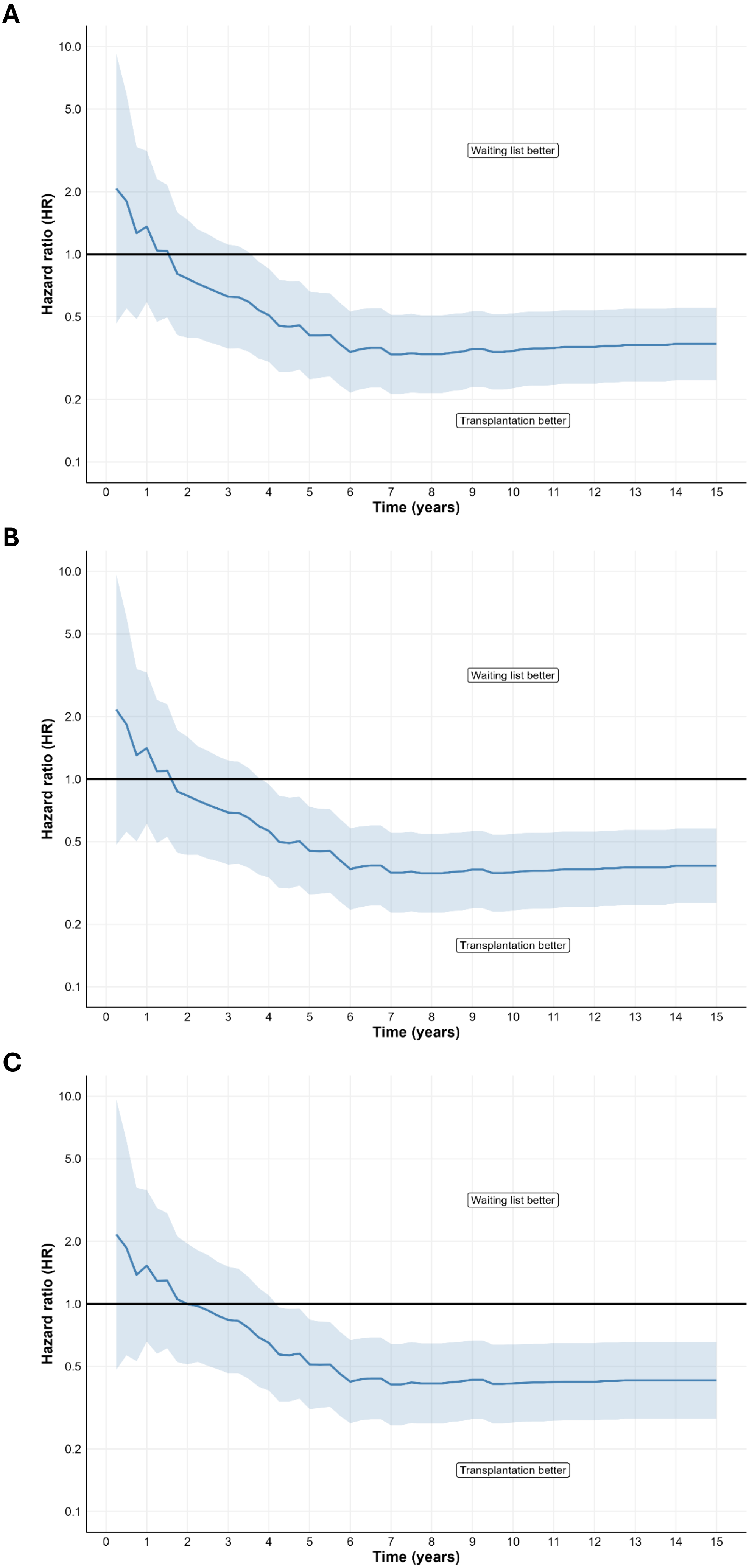
A
B
C
Hazard ratio (HR)
10.0
5.0
2.0
1.0
0.5
0.2
0.1
Waiting list better
Transplantation better
0
1
2
3
4
5
6
7
8
9
10
11
12
13
14
15
Time (years)

Sup. Figure 4

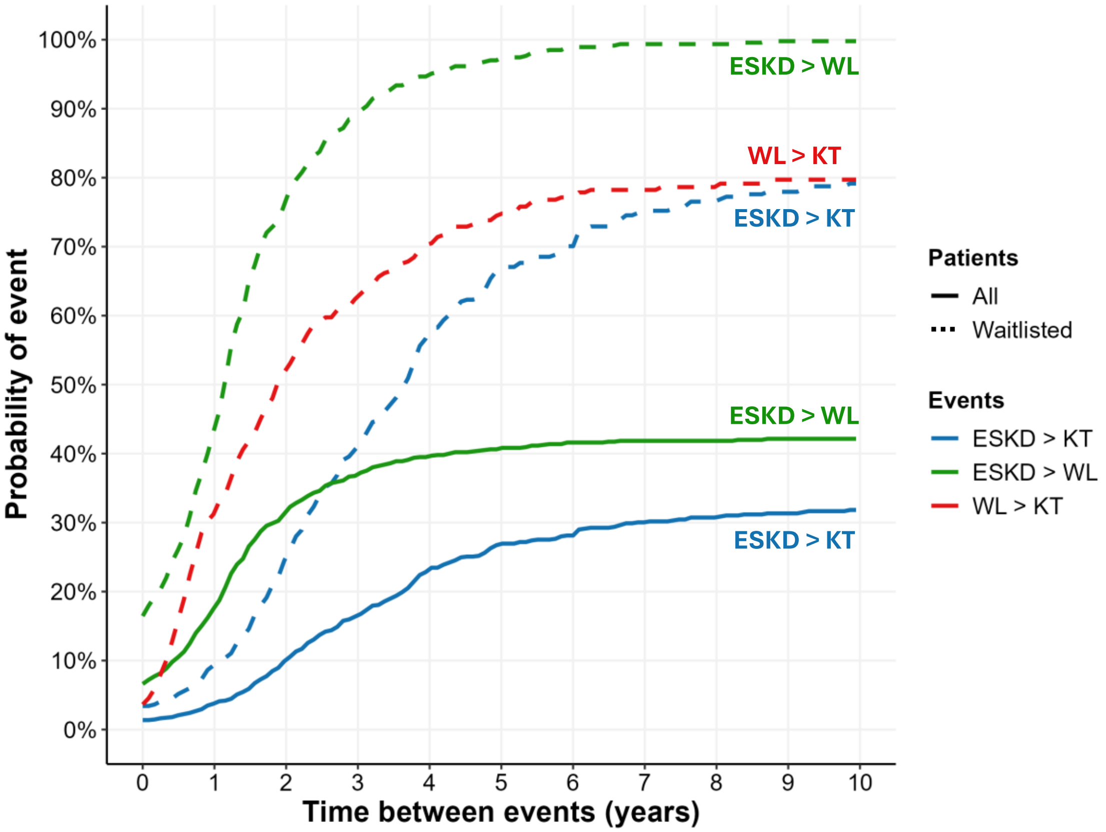

Probability of event
100%
90%
80%
70%
60%
50%
40%
30%
20%
10%
0%
0
1
2
3
4
5
6
7
8
9
10
Time between events (years)
ESKD > WL
WL > KT
ESKD > KT
ESKD > WL
ESKD > KT
Patients
All
Waitlisted
Events
ESKD > KT
ESKD > WL
WL > KT

Supplementary Table S1. Baseline characteristics of AAV-ESKD waitlisted patients according to vasculitis subtype.

* comparison between waitlisted not transplanted and waitlisted transplanted patients.

** Timing between events and delays are shown as median [1st-3rd quartile], in opposition to other continuous data, shown as mean (standard deviation).

"-" means not applicable.

Abbreviations: BMI, body mass index; ESKD, end-stage kidney disease; GPA, granulomatosis with polyangiitis; KT, kidney transplantation; MPA, microscopic polyangiitis.

Supplementary Table S2. Baseline characteristics of AAV-ESKD waitlisted patients according to removal status during follow-up.

"-" means not applicable.

Abbreviations: BMI, body mass index; ESKD, end-stage kidney disease; GPA, granulomatosis with polyangiitis; KT, kidney transplantation; MPA, microscopic polyangiitis.

Supplementary Table S3. Causes of waitlist removal in AAV-ESKD waitlisted, not transplanted patients.

Abbreviations: AAV-ESKD, ANCA-associated vasculitides with end-stage kidney disease.

Supplementary Table S4. Causes of death in AAV-ESKD patients.

* comparison between the 4 categories (waitlisted non-transplanted vs transplanted patients).

** comparison between the 15 categories (waitlisted non-transplanted vs transplanted patients).

Supplementary Table S5. Baseline characteristics of AAV-ESKD waitlisted patients according to date of dialysis initiation (before or after 2012).

"-" means not applicable.

Abbreviations: BMI, body mass index; ESKD, end-stage kidney disease; GPA, granulomatosis with polyangiitis; KT, kidney transplantation; MPA, microscopic polyangiitis.

Supplementary Table S6. Causes of allograft failure in AAV-ESKD transplanted patients.

Abbreviations: AAV-ESKD, ANCA-associated vasculitides with end-stage kidney disease.

Supplementary Table S1

	GPA				MPA			
	**ESKD Waitlisted** N = 242	**Waitlisted Not transplanted** N = 66	**Waitlisted Transplanted** N = 176	**p-value ***	**ESKD Waitlisted** N = 226	**Waitlisted Not transplanted** N = 84	**Waitlisted Transplanted** N = 142	**p-value ***
**Baseline characteristics**								
Male sex	158 (65%)	40 (61%)	118 (67%)	0.3	148 (65%)	63 (75%)	85 (60%)	**0.021**
Age								
at ESKD (years)	57 (11)	60 (10)	56 (12)	**0.025**	57 (14)	60 (11)	55 (15)	**0.009**
at waitlisting (years)	59 (11)	61 (9)	58 (12)	**0.013**	58 (14)	61 (11)	57 (16)	**0.006**
at transplantation (years)	59 (12)	-	59 (12)	-	58 (16)	-	58 (16)	-
First modality				0.055				0.3
Hemodialysis	210 (87%)	61 (92%)	149 (85%)		195 (86%)	76 (90%)	119 (84%)	
Peritoneal dialysis	19 (7.9%)	5 (7.6%)	14 (8.0%)		28 (12%)	8 (9.5%)	20 (14%)	
Pre-emptive transplantation	13 (5.4%)	0 (0%)	13 (7.4%)		3 (1.3%)	0 (0%)	3 (2.1%)	
Calculated Panel Reactive Antibody (%)	14 (27)	21 (33)	11 (23)	0.052	14 (28)	15 (31)	13 (27)	0.6
**Comorbidities**								
Not able to walk alone	4 (1.9%)	1 (1.7%)	3 (2.0%)	>0.9	4 (1.9%)	2 (2.6%)	2 (1.5%)	0.6
Albumin level < 30 g/L	72 (37%)	26 (44%)	46 (34%)	0.2	75 (36%)	27 (36%)	48 (36%)	>0.9
Albumin level < 36 g/L	127 (66%)	46 (78%)	81 (60%)	**0.018**	142 (69%)	53 (71%)	89 (67%)	0.6
BMI				**0.049**				0.4
< 18.5 kg/m²	15 (6.8%)	4 (6.8%)	11 (6.9%)		16 (8.0%)	7 (9.3%)	9 (7.1%)	
18.5 - 25 kg/m²	95 (43%)	18 (31%)	77 (48%)		114 (57%)	38 (51%)	76 (60%)	
> 25 kg/m²	109 (50%)	37 (63%)	72 (45%)		71 (35%)	30 (40%)	41 (33%)	
Diabetus mellitus	37 (16%)	20 (31%)	17 (9.9%)	**<0.001**	33 (15%)	14 (17%)	19 (13%)	0.5
Cardiovascular comorbidities				**0.002**				0.2
None	185 (79%)	41 (64%)	144 (85%)		168 (75%)	62 (75%)	106 (75%)	
Only one	41 (18%)	20 (31%)	21 (12%)		38 (17%)	11 (13%)	27 (19%)	
At least two	8 (3.4%)	3 (4.7%)	5 (2.9%)		18 (8.0%)	10 (12%)	8 (5.7%)	
Respiratory insufficiency	24 (10%)	11 (17%)	13 (7.6%)	**0.031**	28 (13%)	17 (21%)	11 (7.8%)	**0.004**
Cancer	7 (3.2%)	1 (1.6%)	6 (3.8%)	0.7	9 (4.1%)	4 (4.9%)	5 (3.6%)	0.7
**Timings and delays** **								
Time from dialysis to waitlisting (months)	14 (4, 24)	16 (7, 30)	13 (2, 24)	0.083	13 (6, 20)	14 (7, 23)	13 (6, 20)	0.3
Time from dialysis to KT (months)	30 (18, 47)	-	30 (18, 47)	-	33 (21, 47)	-	33 (21, 47)	-
Time from waitlisting to KT (months)	12 (5, 27)	-	12 (5, 27)	-	16 (8, 27)	-	16 (8, 27)	-
Follow-up since dialysis initiation (months)	90 (52, 136)	48 (24, 71)	111 (70, 158)	**<0.001**	64 (34, 111)	34 (20, 60)	92 (52, 127)	**<0.001**
Follow-up since waitlisting (months)	68 (38, 121)	27 (6, 48)	95 (55, 138)	**<0.001**	51 (18, 92)	18 (3, 43)	74 (41, 113)	**<0.001**
Follow-up since transplantation (months)	75 (32, 119)	-	75 (32, 119)	-	52 (18, 91)	-	52 (18, 91)	-
**Transplantation**								
Donor type				**<0.001**				**<0.001**
None	66 (27%)	66 (100%)	-		84 (37%)	84 (100%)	-	
Deceased donor	159 (66%)	-	159 (90%)		123 (54%)	-	123 (87%)	
Living donor	17 (7.0%)	-	17 (9.7%)		19 (8.4%)	-	19 (13%)	
**Outcomes**								
Graft failure	27 (15%)	-	27 (15%)	-	23 (16%)	-	23 (16%)	-
Death	78 (32%)	28 (42%)	50 (28%)	**0.038**	70 (31%)	34 (40%)	36 (25%)	**0.017**

Supplementary Table S2

	**Waitlisted, not removed** N = 434	**Waitlisted, removed** N = 34	**p-value**
**Baseline characteristics**			
Vasculitis subtype			0.6
GPA	226 (52%)	16 (47%)	
MPA	208 (48%)	18 (53%)	
Male sex	283 (65%)	23 (68%)	0.8
Age			
at ESKD (years)	57 (13)	64 (8)	**<0.001**
at waitlisting (years)	58 (13)	65 (8)	**<0.001**
First modality			0.8
Hemodialysis	374 (86%)	31 (91%)	
Peritoneal dialysis	44 (10%)	3 (8.8%)	
Pre-emptive transplantation	16 (3.7%)	0 (0%)	
Calculated Panel Reactive Antibody (%)	14 (27)	21 (34)	0.3
**Comorbidities**			
Not able to walk alone	8 (2.1%)	0 (0%)	>0.9
Albumin level < 30 g/L	138 (37%)	9 (32%)	0.6
Albumin level < 36 g/L	250 (67%)	19 (68%)	>0.9
BMI			0.4
< 18.5 kg/m²	30 (7.7%)	1 (3.4%)	
18.5 - 25 kg/m²	197 (50%)	12 (41%)	
> 25 kg/m²	164 (42%)	16 (55%)	
Diabetus mellitus	58 (14%)	12 (35%)	**<0.001**
Cardiovascular comorbidities			0.6
None	328 (77%)	25 (74%)	
Only one	73 (17%)	6 (18%)	
At least two	23 (5.4%)	3 (8.8%)	
Respiratory insufficiency	46 (11%)	6 (18%)	0.3
Cancer	16 (3.9%)	0 (0%)	0.6

Supplementary Table S3

Cause of waitlist removal	Number of patients
Deterioration of clinical condition (not linked to vasculitis)	20 (58.8%)
Patient decision	8 (23.5%)
Deterioration of clinical condition (linked to vasculitis)	5 (14.7%)
Clinical condition improvement	1 (2.9%)
Total	**34 (100%)**

Supplementary Table S4

Cause of death	Not waitlisted	Waitlisted, not transplanted			Transplanted		
	All deaths N = 508	All deaths N = 62	Deaths during waitlisted period N = 44	Deaths after waitlist removal N = 18	All deaths N = 86	Deaths during transplant period N = 63	Deaths after return to dialysis N = 23
Others	286 (56%)	28 (45%)	20 (45%)	8 (44%)	37 (43%)	28 (44%)	9 (39%)
Infection	110 (22%)	16 (26%)	12 (27%)	4 (22%)	23 (27%)	18 (29%)	5 (22%)
Cancer	30 (5.9%)	9 (15%)	5 (11%)	4 (22%)	16 (19%)	13 (21%)	3 (13%)
Cardiovascular	82 (16%)	9 (15%)	7 (16%)	2 (11%)	10 (12%)	4 (6.3%)	6 (26%)

p-value * = 0.9

Cause of death (detailed)	Not waitlisted	Waitlisted, not transplanted			Transplanted		
	All deaths N = 508	All deaths N = 62	Deaths during waitlisted period N = 44	Deaths after waitlist removal N = 18	All deaths N = 86	Deaths during transplant period N = 63	Deaths after return to dialysis N = 23
Other known cause	85 (17%)	9 (15%)	8 (18%)	1 (5.6%)	17 (20%)	16 (25%)	1 (4.3%)
Unknown cause	101 (20%)	12 (19%)	10 (23%)	2 (11%)	14 (16%)	11 (17%)	3 (13%)
Rapid or unexpected death, shock without precision	47 (9.3%)	5 (8.1%)	0 (0%)	5 (28%)	2 (2.3%)	0 (0%)	2 (8.7%)
Cachexia	53 (10%)	1 (1.6%)	1 (2.3%)	0 (0%)	3 (3.5%)	1 (1.6%)	2 (8.7%)
Hyperkaliemia	0 (0%)	0 (0%)	0 (0%)	0 (0%)	1 (1.2%)	0 (0%)	1 (4.3%)
Liver disease	0 (0%)	1 (1.6%)	1 (2.3%)	0 (0%)	0 (0%)	0 (0%)	0 (0%)
Infection	110 (22%)	16 (26%)	12 (27%)	4 (22%)	23 (27%)	18 (29%)	5 (22%)
Cancer	30 (5.9%)	9 (15%)	5 (11%)	4 (22%)	16 (19%)	13 (21%)	3 (13%)
Other cardiovascular cause	21 (4.1%)	5 (8.1%)	4 (9.1%)	1 (5.6%)	3 (3.5%)	0 (0%)	3 (13%)
Heart failure	22 (4.3%)	0 (0%)	0 (0%)	0 (0%)	3 (3.5%)	2 (3.2%)	1 (4.3%)
Cerebrovascular disease	19 (3.7%)	0 (0%)	0 (0%)	0 (0%)	3 (3.5%)	2 (3.2%)	1 (4.3%)
Myocardial infarction	4 (0.8%)	2 (3.2%)	2 (4.5%)	0 (0%)	0 (0%)	0 (0%)	0 (0%)
Other ischemic cardiopathy	4 (0.8%)	1 (1.6%)	0 (0%)	1 (5.6%)	0 (0%)	0 (0%)	0 (0%)
Pulmonary embolism	4 (0.8%)	1 (1.6%)	1 (2.3%)	0 (0%)	0 (0%)	0 (0%)	0 (0%)
Rhythm disorder	8 (1.6%)	0 (0%)	0 (0%)	0 (0%)	1 (1.2%)	0 (0%)	1 (4.3%)

p-value ** = 0.2

Supplementary Table S5

	**Waitlisted before 2012** N = 142	**Waitlisted after 2012** N = 326	**p-value**
**Baseline characteristics**			
Vasculitis subtype			**0.002**
GPA	89 (63%)	153 (47%)	
MPA	53 (37%)	173 (53%)	
Male sex	90 (63%)	216 (66%)	0.5
Age			
at ESKD (years)	55 (13)	58 (13)	**0.044**
at waitlisting (years)	57 (13)	59 (13)	0.094
at transplantation (years)	57 (14)	60 (14)	0.2
First modality			0.7
Hemodialysis	126 (89%)	279 (86%)	
Peritoneal dialysis	12 (8.5%)	35 (11%)	
Pre-emptive transplantation	4 (2.8%)	12 (3.7%)	
Calculated Panel Reactive Antibody (%)	18 (31)	13 (27)	0.2
**Comorbidities**			
Not able to walk alone	3 (2.6%)	5 (1.6%)	0.5
Albumin level < 30 g/L	43 (40%)	104 (35%)	0.4
Albumin level < 36 g/L	72 (67%)	197 (67%)	>0.9
BMI			0.6
< 18.5 kg/m²	11 (9.6%)	20 (6.6%)	
18.5 - 25 kg/m²	57 (50%)	152 (50%)	
> 25 kg/m²	47 (41%)	133 (44%)	
Diabetus mellitus	11 (8.1%)	59 (18%)	**0.007**
Cardiovascular comorbidities			>0.9
None	104 (77%)	249 (77%)	
Only one	23 (17%)	56 (17%)	
At least two	8 (5.9%)	18 (5.6%)	
Respiratory insufficiency	13 (9.6%)	39 (12%)	0.4
Cancer	2 (1.5%)	14 (4.5%)	0.2
**Transplantation**			
Donor type			0.2
Deceased donor	110 (92%)	172 (87%)	
Living donor	10 (8.3%)	26 (13%)	

Supplementary Table S6

Cause of allograft failure	Number of patients
Rejection	23 (46%)
Hyperacute	1 (2%)
Acute	5 (10%)
Chronic	17 (34%)
Vascular complications	11 (22%)
Vasculitis relapse	4 (8%)
Chronic kidney disease	3 (6%)
Infections	3 (6%)
Sepsis	2 (4%)
Viral infection (excluding viral hepa	1 (2%)
Heart failure	1 (2%)
Other	5 (10%)
Total	**50 (100%)**
